# Computational genome-wide identification of heat shock protein genes in the bovine genome

**DOI:** 10.12688/f1000research.16058.1

**Published:** 2018-09-20

**Authors:** Oyeyemi O. Ajayi, Sunday O. Peters, Marcos De Donato, Sunday O. Sowande, Fidalis D.N. Mujibi, Olanrewaju B. Morenikeji, Bolaji N. Thomas, Matthew A. Adeleke, Ikhide G. Imumorin

**Affiliations:** 1 Department of Animal Breeding and Genetics, Federal University of Agriculture, Abeokuta, Nigeria; 2 International Programs, College of Agriculture and Life Sciences, Cornell University, Ithaca, NY, 14853, USA; 3 Department of Animal Science, Berry College, Mount Berry, GA, 30149, USA; 4 Departamento Regional de Bioingenierias, Tecnologico de Monterrey, Escuela de Ingenieria y Ciencias, Queretaro, Mexico; 5 Department of Animal Production and Health, Federal University of Agriculture, Abeokuta, Nigeria; 6 Usomi Limited, Nairobi, Kenya; 7 Department of Animal Production and Health, Federal University of Technology, Akure, Nigeria; 8 Department of Biomedical Sciences, Rochester Institute of Technology, Rochester, NY, 14623, USA; 9 School of Life Sciences, University of KwaZulu-Natal, Durban, 4000, South Africa; 10 School of Biological Sciences, Georgia Institute of Technology, Atlanta, GA, 30032, USA; 11 African Institute of Bioscience Research and Training, Ibadan, Nigeria

**Keywords:** Cattle, bovine genome, heat shock proteins, Hsp genes, molecular chaperones

## Abstract

**Background:** Heat shock proteins (HSPs) are molecular chaperones known to bind and sequester client proteins under stress.

**Methods:** To identify and better understand some of these proteins, we carried out a computational genome-wide survey of the bovine genome. For this, HSP sequences from each subfamily (sHSP, HSP40, HSP70 and HSP90) were used to search the Pfam (Protein family) database, for identifying exact HSP domain sequences based on the hidden Markov model. ProtParam tool was used to compute potential physico-chemical parameters detectable from a protein sequence. Evolutionary trace (ET) method was used to extract evolutionarily functional residues of a homologous protein family.

**Results:** We computationally identified 67 genes made up of 10, 43, 10 and 4 genes belonging to small HSP, HSP40, HSP70 and HSP90 families respectively. These genes were widely dispersed across the bovine genome, except in chromosomes 24, 26 and 27, which lack bovine HSP genes. We found an uncharacterized outer dense fiber ( *ODF1*) gene in cattle with an intact alpha crystallin domain, like other small HSPs. Physico-chemical characteristic of aliphatic index was higher in HSP70 and HSP90 gene families, compared to small HSP and HSP40. Grand average hydropathy showed that small HSP (sHSP), HSP40, HSP70 and HSP90 genes had negative values except for *DNAJC22*, a member of HSP40 gene family. The uniqueness of *DNAJA3* and *DNAJB13* among HSP40 members, based on multiple sequence alignment, evolutionary trace analysis and sequence identity dendrograms, suggests evolutionary distinct structural and functional features, with unique roles in substrate recognition and chaperone functions. The monophyletic pattern of the sequence identity dendrograms of cattle, human and mouse HSP sequences suggests functional similarities.

**Conclusions:** Our computational results demonstrate the first-pass *in-silico* identification of heat shock proteins and calls for further investigation to better understand their functional roles and mechanisms in Bovidae.

## Introduction

Most newly synthesized proteins require the interplay of evolutionarily conserved protein co-factors known as molecular chaperones, activated in response to heat stress or other chemical stressors that impair cellular activity. Organisms respond to environmental stress through reprogramming leading to the production of heat shock proteins (HSPs) ( Hartl & Hayer-Hartl, 2002). Effects of HSP production include maintaining cellular protein homeostasis and guiding against cellular dysfunction, increased responsiveness to stress insults, microfilament stabilization, etc. ( Kregel, 2002). In addition, HSP70s and HSP40s synergistically suppress the formation of toxic proteins that drive neurodegeneration ( Meriin *et al.*, 2002).

HSPs are classified into six main families (small HSPs, HSP40, HSP60, HSP70, HSP90 and HSP110), based on molecular mass ( Johnson *et al.*, 2003; Kappe *et al.*, 2002). In addition, individual families also have subfamily differentiations, all contributing to specific functions in eukaryotes ( Cheetham & Caplan, 1998). HSP40 family is grouped into three subtypes, based on the extent of domain conservation when compared to the *Escherichia coli* gene *dnaJ* ( Cheetham & Caplan, 1998). HSP70s are highly conserved across many phyla, with distinctive N- and C-terminal domains interacting in an allosteric fashion ( Craig *et al.*, 2006). HSP90 genes (inducible HSP-α, and constitutive HSP-β) ( Csermely *et al.*, 1998; Hoffman & Hovemann, 1988), although located in the nucleus, express their protein function in the cytosol, endoplasmic reticulum, chloroplast and mitochondria ( Emelyanov, 2002; Krishna & Gloor, 2001; Stechmann & Cavalier-Smith, 2004). The first step towards a better understanding of bovine HSPs require knowledge of the actual number of HSP genes in cattle. In this study, we identified the number, chromosomal locations and the physico-chemical properties of 67 HSP genes in the bovine genome. Evolutionarily conserved and class specific residues were inferred using evolutionary trace (ET) analysis and sequence identity dendrograms were constructed using human, mouse and cattle HSP sequences to infer functional similarity among these three species. Our results contribute to the biology of HSPs in cattle, possible application in animal breeding and further clarifies intercontinental bovine adaptation mechanisms.

## Methods

### Identification of HSPs in cattle

We identified all putative heat shock protein genes at the genome-wide level in cattle using published human and mouse sequences as queries. Due to the variation in HSP gene family sequences, we used three representative HSP sequences from each subfamily (sHSP, HSP40, HSP70 and HSP90) to search the Pfam (Protein family) database, for identifying exact HSP domain sequences based on the hidden Markov model (HMM) ( Finn *et al.*, 2010). The 12 query sequences are as follows: human sHSPs; NP_001531.1, NP_1499971.1 and mouse Hsp; NP_034094.1; human HSP40; NP_001530.1, NP699161.1 and mouse HSP40: NP033610.1; human HSP70 NP_005336, NP_002146.2 and mouse Hsp70 NP_084477.1; human HSP90; NP_001017963 and NP_003290.1 and mouse NP_032328.2. Pfam domain PF00011.16 (Hsp20 domain), PF00226.26 (DNA J domain), PF00012.15 (Hsp70 domain) and PF00183.13 (Hsp90 domain) were used to carry out a protein-protein BLAST search ( *p* value = 0.001) of the non-redundant protein sequences in *Bos taurus* using the BLOSUM62 matrix, with an expected threshold of 10, a word size of 6, a gap cost of 11 with an extension 1, and with a conditional compositional score matrix adjustment. We acquired the starting chromosomal locations of candidate HSP genes searching through TBLASTN (p value = 0.001) in the non-redundant protein sequences for *Bos taurus* using the BLOSUM62 matrix, with an expected threshold of 10, a word size of 6, a gap cost of 11 with an extension 1, and with a conditional compositional score matrix adjustment and filtering the low complexity regions. BLAT searches of the UCSC database were used to confirm the chromosomal locations of the HSP sequences. Redundant sequences similarly located chromosomally were rejected. Candidate sequences fitting our criteria were analyzed in the Pfam database and detected using the SMART program (version 6), as described ( Letunic *et al.*, 2009).

### Characterization of physico-chemical properties

We utilized the ProtParam tool (Swiss Institute of Bioinformatics) to compute potential physico-chemical parameters detectable from a protein sequence (molecular weight, theoretical isoelectric point (pI), amino acid composition, estimated half-life, aliphatic index, grand average of hydropathicity (GRAVY)) etc., as described ( Gasteiger *et al.*, 2003). All HSP sequences were submitted to this tool for physico-chemical characterization.

### Multiple sequence alignment

Multiple alignments of HSP protein sequences from human, mouse and cattle were performed using ClustalW (version 2). Conserved regions in the alignment were shaded black and less conserved regions were shaded gray ( Gasteiger *et al.*, 2003).

### Evolutionary trace analysis

To extract evolutionarily functional residues of a homologous protein family, we utilized the evolutionary trace (ET) method, as previously described. Multiple sequence alignment obtained from ClustalW for sHSP, HSP40 (type I and II), HSP70 and HSP90 were submitted to ET analysis Web server ( Lichtarge *et al.*, 1996), using input Protein Data Bank (PDB) files 2WJ7 (human alphaB crystallin), 1HDJ (NMR solution structure of the HDJ-1-J-domain), 1YUW (Crystal structure of bovine hsc70 (aa1-554) E213A/D214A mutants, 3Q6N (hexameric structures of Human HSP90) obtained from PDB as trace-to-structure mapping for sHSP, HSP40, HSP70 and HSP90, respectively.

## Results

Our exhaustive search for HSP genes in the bovine genome using human and mouse sequences as queries resulted in the identification of 10 genes belonging to small HSPs ( Table 1), 43 genes belonging to HSP40 gene family ( Table 2), 10 genes belonging to the HSP70 gene family ( Table 3) and 4 genes sharing the HSP90 family ( Table 4). We classified a gene as belonging to sHSP gene family if it contains one or more intact alpha-crystallin domain. In addition to the list of sHSP genes ( Table 1), outer dense fiber protein 1 ( *ODF1*) was identified in the genome-wide search due to the presence of an intact alpha-crystallin domain, as confirmed by Pfam and SMART tools. sHSPs were scattered across the chromosomes, with two genes ( *HSPB2* and *CRYAB*), found on chromosome 15 observed to be approximately 5 kb apart, possibly indicative of a tandem duplication.

**Table 1.** Biochemical characterization of the bovine small heat shock protein genes.

Number	Gene Symbol	GenBank accession number	Size (aa)	MW (Da)	pl	Chromosome	AI	II	GRAVY
1	*HSPB1*	NP_001020740.1	204	22679.3	5.77	25	69.36	59.53	-0.597
2	*HSPB2*	NP_001091850.1	182	20130.3	5.07	15	77.69	45.88	-0.537
3	*HSPB3*	NP_001040035.1	149	16722.2	5.33	20	96.91	46.37	-0.205
4	*HSPB6*	NP_001069495.1	164	17468.9	5.95	18	90.55	60.43	-0.100
5	*HSPB7*	XP_003585853.1	169	18420.4	5.60	2	60.71	60.85	-0.492
6	*HSPB8*	NP_001014955.1	196	21673.4	5.13	17	59.13	72.61	-0.549
7	*HSPB9*	NP_001035667.1	157	16783.9	8.22	19	67.71	47.07	-0.373
8	*CRYAA*	NP_776714.1	173	19790.1	5.78	1	72.08	57.45	-0.494
9	*CRYAB*	NP_776715.1	175	20036.7	6.76	15	77.43	50.92	-0.507
10	*ODF1*	NP_776556.3	262	29419.4	8.40	14	68.44	69.14	-0.259

aa, amino acids; MW, molecular weight; pI, isoelectric point; AI, aliphatic index; II, instability index; GRAVY, grand average of hydropathicity index.

**Table 2.** Biochemical characterization of the bovine heat shock protein 40 protein genes.

Number	Gene symbol	Accession number	Size (aa)	MW (Da)	pl	Chromosome	AI	II	GRAVY
1	*DNAJA1*	NP_001015637.1	397	44960.3	6.71	8	71.89	33.92	-0.719
2	*DNAJA2*	NP_001035581.1	412	45761.6	6.06	18	68.08	39.03	-0.699
3	*DNAJA3*	NP_001073739.1	453	49253.3	9.53	25	67.57	43.48	-0.459
4	*DNAJA4*	NP_001095590.2	426	47972.8	6.63	21	74.77	37.74	-0.726
5	*DNAJB1*	NP_001028935.1	340	38217.3	8.75	7	63.35	49.95	-0.750
6	*DNAJB2*	NP_001029764.1	278	30706.7	5.08	2	56.19	55.94	-0.682
7	*DNAJB3*	NP_001069384.1	244	26914.8	6.07	3	51.56	50.26	-0.657
8	*DNAJB4*	NP_0011039968.1	337	37850.8	8.66	3	63.38	43.18	-0.709
9	*DNAJB5*	NP_001014959.1	348	39075.4	9.12	8	68.07	46.13	-0.673
10	*DNAJB6*	NP_776957.2	242	26943.7	6.99	4	47.15	39.38	-0.733
11	*DNAJB7*	NP_001093200.1	304	35007.2	5.56	5	46.18	40.16	-1.087
	*DNAJB8*	XP_002697194.1	231	25174.8	6.46	22	49.00	46.59	-0.676
12	*DNAJB9*	NP_001179968.1	223	25715.5	7.79	22	41.61	36.63	-0.841
13	*DNAJB11*	NP_001029440.1	358	40503.9	5.92	4	80.25	37.41	-0.568
14	*DNAJB12*	NP_001017946.1	370	41340.2	8.79	1	66.51	39.29	-0.729
15	*DNAJB13*	NP_001029708.1	316	36077.2	7.66	13	81.42	36.59	-0.517
16	*DNAJB14*	NP_001069599.1	379	42487.6	8.69	15	63.59	35.49	-0.840
17	*DNAJC1*	XP_003586814.1	584	67279.8	8.75	6	84.67	61.54	-0.752
18	*DNAJC2*	NP_001068805.1	621	71786.1	8.80	13	60.72	52.57	-1.066
19	*DNAJC3*	NP_777181.1	504	57704.2	5.60	12	78.25	44.89	-0.677
20	*DNAJC4*	XP_002699374.1	235	26860.5	10.65	12	65.70	68.03	-0.859
21	*DNAJC5*	NP_776958.2	198	22132.7	4.93	29	65.15	34.45	-0.4776
22	*DNAJC6*	NP_777261.1	630	69029.9	6.26	13	72.41	53.24	-0.381
23	*DNAJC7*	NP_001192905.1	494	56353.6	6.38	19	64.76	41.07	-0.714
24	*DNAJC8*	XP_002685798.2	253	29822.6	9.04	3	57.08	52.19	-1.322
25	*DNAJC9*	NP_001192615.1	260	30205.0	5.56	28	72.38	43.52	-0.935
26	*DNAJC10*	NP_001092591.1	793	91149.7	6.48	2	77.11	36.43	-0.411
27	*DNAJC11*	NP_001039458.1	559	63237.0	8.50	16	89.92	47.48	-0.405
28	*DNAJC12*	NP_776521.1	198	23268.8	5.11	28	55.20	73.99	-1.067
29	*DNAJC13*	NP_001107217.1	2243	254439.7	6.35	1	92.55	42.29	-0.236
30	*DNAJC14*	NP_776699.1	699	78178.3	8.21	5	66.88	50.72	-0.628
31	*DNAJC15*	NP_001073801.1	149	15850.2	10.10	12	86.64	49.31	-0.200
32	*DNAJC16*	NP_001094561.1	782	90291.0	6.62	16	86.01	40.99	-0.337
33	*DNAJC17*	NP_001039741.1	304	34572.1	7.75	10	78.39	46.79	-0.904
34	*DNAJC18*	NP_001015649.1	357	41545.7	8.25	7	65.88	41.78	-0.875
35	*DNAJC19*	NP_001029630.1	116	12498.6	10.10	1	80.95	43.73	-0.229
36	*DNAJC21*	NP_001179147.1	533	61717.2	5.46	10	52.78	52.93	-1.251
37	*DNAJC22*	NP_001069169.2	347	39014.3	9.47	5	100.92	35.29	0.163
38	*DNAJC24*	NP_001071570.1	149	17135.3	4.61	15	78.59	62.74	-0.541
39	*DNAJC25*	NP_001191924.1	359	42591.0	9.21	8	82.90	61.98	-0.627
40	*DNAJC27*	NP_001091578.1	273	30759.1	8.55	11	75.35	32.99	-0.430
41	*DNAJC30*	NP_001074386.1	226	25692.1	10.44	25	68.32	53.42	-0.742
42	*SAMD13*	NP_001073112.1	195	21985.6	9.20	3	59.44	43.31	-0.675
43	*SEC63*	NP_001179744.1	760	87882.7	5.20	9	79.14	51.48	-0.685

aa, amino acids; MW, molecular weight; pI, isoelectric point; AI, aliphatic index; II, instability index; GRAVY, grand average of hydropathicity index.

**Table 3.** Biochemical characterization of the bovine heat shock protein 70 protein genes.

Number	Gene symbol	Genbank accession number	Size (aa)	MW (Da)	pl	Chromosome	AI	II	GRAVY
1	*HSPA1A*	NP_976067.3	641	70250.4	5.55	23	85.07	32.35	-0.399
2	*HSPA1L*	NP_001161367.1	641	70389.0	5.89	23	85.99	32.93	-0.347
3	*HSPA2*	NP_776769.1	631	69199.1	5.32	10	82.85	34.40	-0.445
4	*HSPA4*	NP_001107664.1	840	94509.3	5.13	7	76.01	45.24	-0.556
5	*HSPA5*	NP_001068616.1	655	72400.0	5.07	11	86.46	32.52	-0.477
6	*HSPA6*	XP_002685896.1	643	70956.0	5.73	3	83.89	39.53	-0.404
7	*HSPA8*	NP_776770.2	650	71270.5	5.37	15	80.86	37.59	-0.458
8	*HSPA9*	NP_001029696.1	679	73741.5	5.97	7	82.34	37.26	-0.400
9	*HSPA13*	NP_001033594.1	471	51920.4	5.18	1	97.64	33.27	-0.130
10	*HSPA14*	NP_001039853.1	509	54929.6	5.58	13	94.46	44.95	-0.095

aa, amino acids; MW, molecular weight; pI, isoelectric point; AI, aliphatic index; II, instability index; GRAVY, grand average of hydropathicity index.

**Table 4.** Biochemical characterization of the bovine heat shock protein 90 protein genes.

Number	Gene Symbol	Accession Number	Size (aa)	MW (Da)	pl	Chromosome	AI	II	GRAVY
1	*HSP90AA1*	NP_001012688.1	703	84730.7	4.92	21	79.40	42.65	-0.751
2	*HSP90AB1*	NP_001073105.1	724	83253.1	4.96	23	81.05	42.30	-0.679
3	*HSP90B1*	NP_777125.1	804	92426.7	4.76	5	76.63	40.41	-0.722
4	*TRAP1*	NP_001033764	703	79380.7	6.66	25	90.36	43.55	-0.316

aa, amino acids; MW, molecular weight; pI, isoelectric point; AI, aliphatic index; II, instability index; GRAVY, grand average of hydropathicity index.

The 43 genes belonging to the HSP40 gene family were sub-classified depending on domain conservation into types I, II and III. A total of four HSP40 genes ( *DNAJA1*, *DNAJA2*, *DNAJA3* and *DNAJA4*) ( Table 2) possessed the characteristic four canonical domains: J, Glycine-phenylalanine(G/F)-rich region, 2 zinc-finger like motifs, and the carboxyl-terminal (CTD), first observed in *E coli*. In total, 12 HSP40 genes were observed to belong to HSP40 type II (lack zinc-finger-like motifs), and 27 HSP40 genes were assigned to HSP40 type III due to the presence of a single J domain. Although 22 out of 29 chromosomes contained the hsp40 gene family, with the highest number of HSP40 genes found on chromosome 12 ( Table 2), no HSP40 gene was found on chromosomes 14, 17, 20, 23, 26 and 27.

A total of 10 HSP70 genes along with their chromosomal positions are presented in Table 3. HSP 70-1a, hsp70-1b and hsp70-1L were mapped to chromosome 23 with less than 1 kb between these genes. However, HSP 70-1a and hsp70-1b were mapped to the same region due to high sequence identity (99%) between these two genes. Similarly, four HSP90 genes in cattle were assigned to hsp90 gene family; and *HSP90AA1*, *HSP90AB1*, *HSP90B1* and *TRAP1* were mapped to chromosome 21, 23, 5 and 25, respectively ( Table 4). Jointly considering the chromosomal locations of all the HSP genes in this study, chromosomes 24, 26 and 27 completely lack HSP genes in the bovine genome.

### Physico-chemical characterization of bovine HSP sequences

The physico-chemical parameters indicated that in sHSPs, pI ranged from 5.07 to 8.40, with most members of the sHSP being acidic except for HSPB9 and ODF-1 which are basic in nature ( Table 1). pI for HSP40 proteins in the gene family ranged from 4.61 in DNAJC24 to 10.65 in DNAJC4, with others nestled between these two extremes ( Table 2). However, when jointly considered, 20 HSP40 proteins were acidic while 23 were basic. In the HSP70 family, all members were acidic in nature, with values ranging from 5.07 (HSPA5) to 5.97 (HSPA9) ( Table 3). Similar results were obtained for members of HSP90 family, with HSP90B1 having the lowest pI (4.76) while TRAP1 has the highest value (6.66) ( Table 4).

Results obtained for the instability index revealed that all sHSP and HSP90 family members were unstable (II>40), with majority of the HSP40 and HSP70 families very stable (II<40). Comparatively, values obtained for aliphatic index were high among sHSP, HSP40, HSP70 and HSP90 families, with higher values more pronounced in the HSP70 and HSP90 families. Similarly, results obtained from GRAVY showed sHSPs, HSP40, HSP70 and HSP90 proteins with negative values, except for DNAJC22 ( Table 2), which was observed to be positive.

### Multiple sequence alignment of bovine HSP sequences

**Figure 1.**
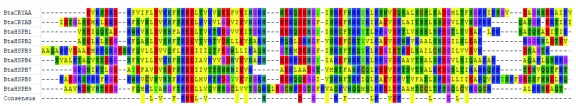
Multiple sequence alignment of cattle heat shock protein 20 (HSP20) domains, excluding ODF1, a more diverse member of HSP20 gene family. The amino acid residues evolutionarily conserved are shown in as the consensus at the bottom. The sites with amino acids that have the same biochemical characteristics are shown as colored boxes. Bta, *Bos taurus*.

**Figure 2.**
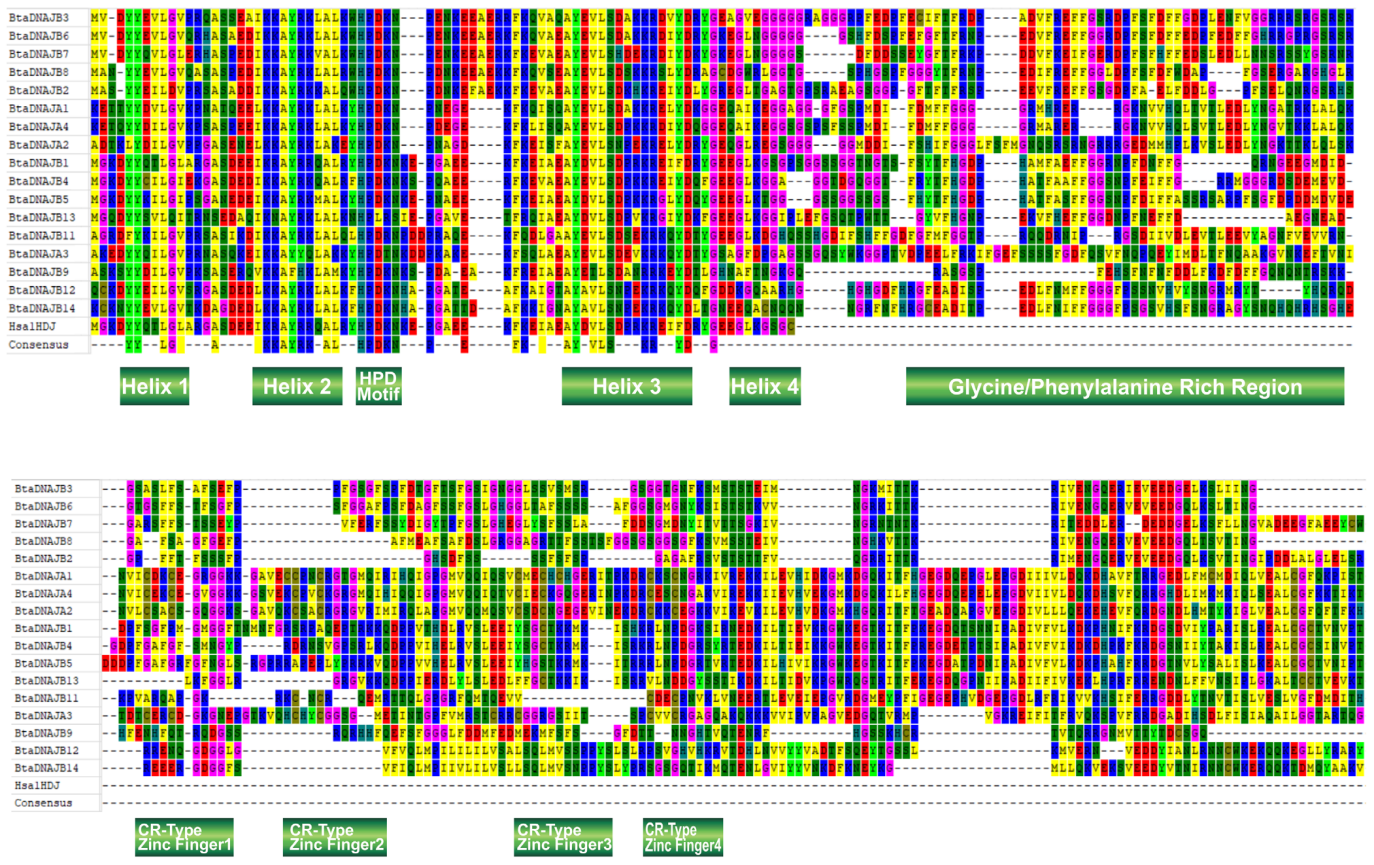
Multiple sequence alignment of cattle Type 1 and 2 heat shock protein 40 domains, including the human 1HDJ domain. The J domain is the most conserved (top region). The 4 conserved CR-type Zinc finger, characterized by the CxxCxGxG motifs, and the glycine/phenyl alanine region are also shown. The amino acid residues evolutionarily conserved are shown in as the consensus at the bottom. The sites with amino acids having the same biochemical characteristics are shown as colored boxes. Bta represents *Bos taurus*, and Hsa *Homo sapiens*.

Alignment results for sHSP gene family in cattle showed that the alpha crystallin domain is much more highly conserved than the N- and C-terminal regions. Multiple sequence alignment identified phenylalanine (F), proline (P), glycine (G), leucine (L), glycine (G) and L to be evolutionarily conserved ( Figure 1). Interestingly, all these residues were found to reside in the alpha crystallin domain, which may indicate their functional importance. Type 1 and II HSP40 gene family protein sequences and the NMR solution structure of human HSP40 (1hdj), containing only the J domain, were aligned. Amino residues tyrosine (Y), L (present in helix 1); lysine (K), alanine (A), A (present in helix 2); H, P (present in the HPD loop); F, A, Y, L (present in helix 3); serine (S), arginine (R), aspartic acid (D) (present in helix 4), and G were observed to be evolutionarily conserved among type I and II members; more importantly was the fact that all these conserved residues were localized in the J domain of Hsp40 genes. The sequence motif CxxCxGxG, a characteristic of the zinc finger domain was only observed in the HSP40 type 1 gene family ( Figure 2).

### Evolutionary trace analysis and sequence identity dendrogram among human, mouse and cattle sequences

**Figure 3.**
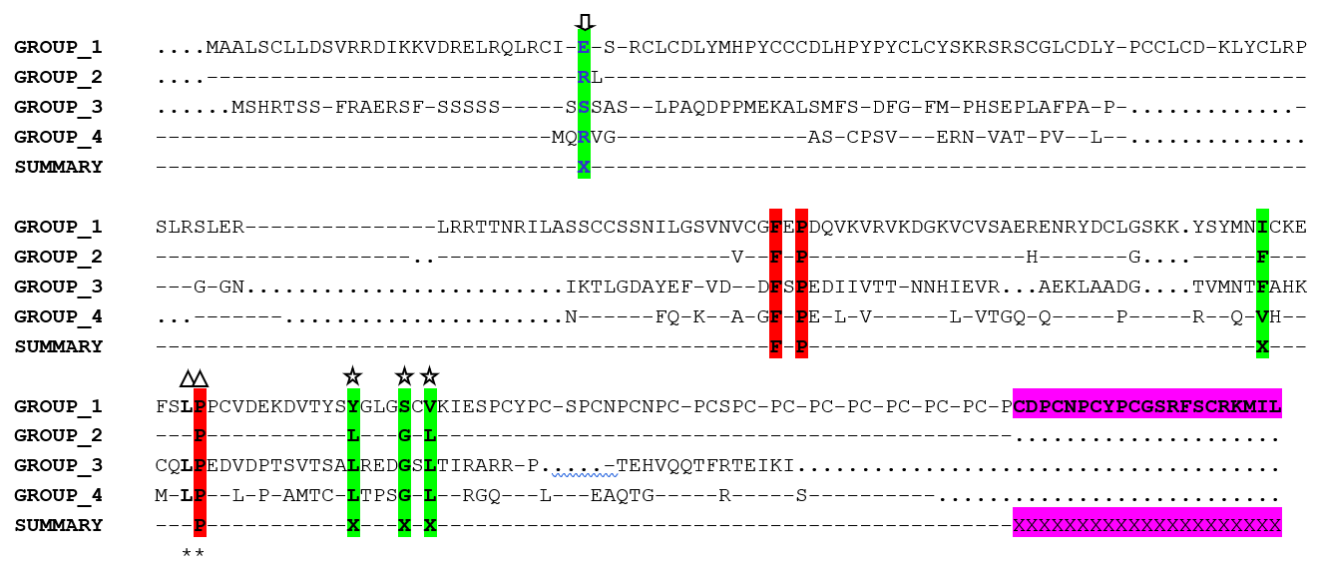
Evolutionary trace analysis of the small heat shock protein (sHSP) gene family in human, mouse and cattle. Every member of sHSPs was partitioned into four different groups based on the degree of conservation. Group 1 consists of human, mouse and cattle ODF1 protein. Group 2 consists of HSPB3, HSPB2, CRYAA, CRYAB, HSPB6, HSPB1 and HSPB8; Group 3 consists of HSPB7 while Group 4 consists of HSPB9. Conserved residues were colored red while class specific residues were colored green. A class specific residue was observed in the N terminal domain conferring group specificity to chaperoning functions (indicated by an arrow). Amino acid residues with triangle and star in the figure above have been identified elsewhere ( Laksanalamai & Robb, 2004). The presence of an extra 21 amino acid residues in ODF1 gene which is lacking in other sHSP members is highlighted in magenta coloration. The sign “…..” represents the presence of invariant residue in each group while the sign “----” represents the absence of a residue at that position.

To examine the structural context of the invariant residues in sHSP gene family, the human alpha B crystallin (2WJ7) was used as our reference structure. sHSP sequences of human, mouse and cattle utilized for evolutionary trace analysis were partitioned into four groups. Multiple sequence alignment of the consensus sequences obtained from conserved residues in each group resulted in a trace, which identified three evolutionarily functional residues (F, P, P) and five residues which appeared to be class-specific ( Figure 3). Amino acid residues isoleucine (I) and valine (V), were class-specific to group 1 (ODF1) and group IV (HSPB9), respectively, while F was specific to groups 2 and 3. In addition, while the second, third and fourth-class specific residues Y, S and V were observed in group 1, residues L and G were observed to be peculiar to groups 2, 3 and 4 respectively. Interestingly, most of these evolutionarily conserved and class-specific residues were found in the alpha-crystallin domain, apart from a class-specific residue found in the N-terminal region (indicated by an arrow in Figure 3).

**Figure 4.**
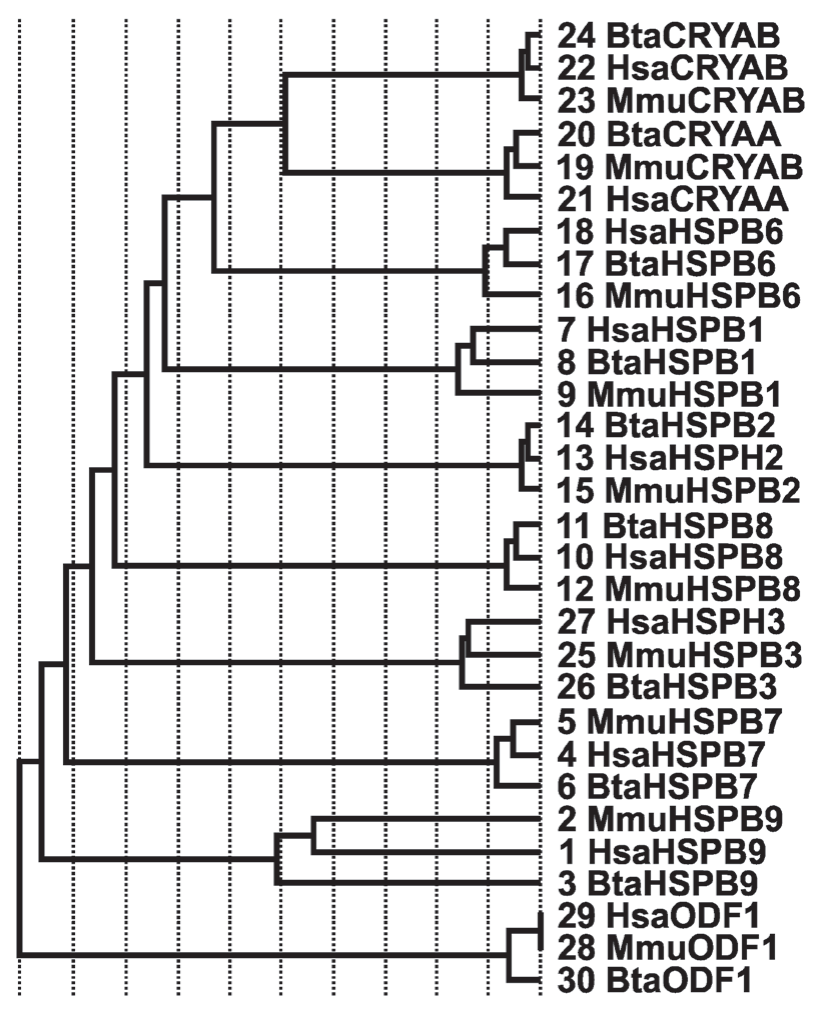
Sequence identity dendrogram of small heat shock proteins. The first three letters Hsa, Bta and Mmu corresponds to human, bovine and mouse, respectively, followed by the gene names. The numbers represent the sequence numbers used in the evolutionary trace analysis.

**Figure 5.**
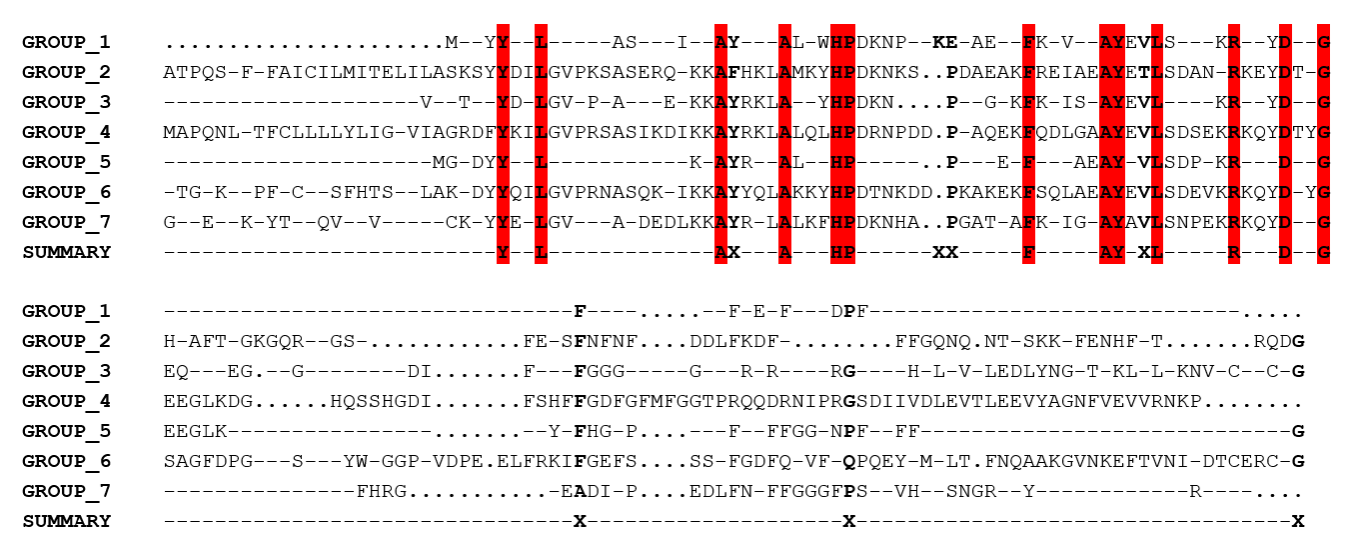
Evolutionary trace analysis of the heat shock protein 40 (HSP40) gene family in human, mouse and cattle (J domain region only). Every member of HSP40 gene family were partitioned into seven different groups based on the degree of conservation. All groups consisted of human, mouse and cattle sequences. Group 1 consists of DNAJB2, DNAJB3, DNAJB6, DNAJB7 and DNAJB8. Group 2 consist of only DNAJB9; Group 3 consists of DNAJA1, DNAJA2, and DNAJA4; Group 4 consists of DNAJB11; Group 5 consists of DNAJB1, DNAJB 4, DNAJB 5 and DNAJB 13; Group 6 consist only DNAJA3 while Group 7 consist of DNAJB12 and DNAJB14. Conserved residues were colored red and they were all localized in the J domain while class specific residues were denoted by the sign “X”. The symbol “….” Represents the presence of invariant residue in each group while the symbol “----” represents the absence of a residue at that position.

**Figure 6.**
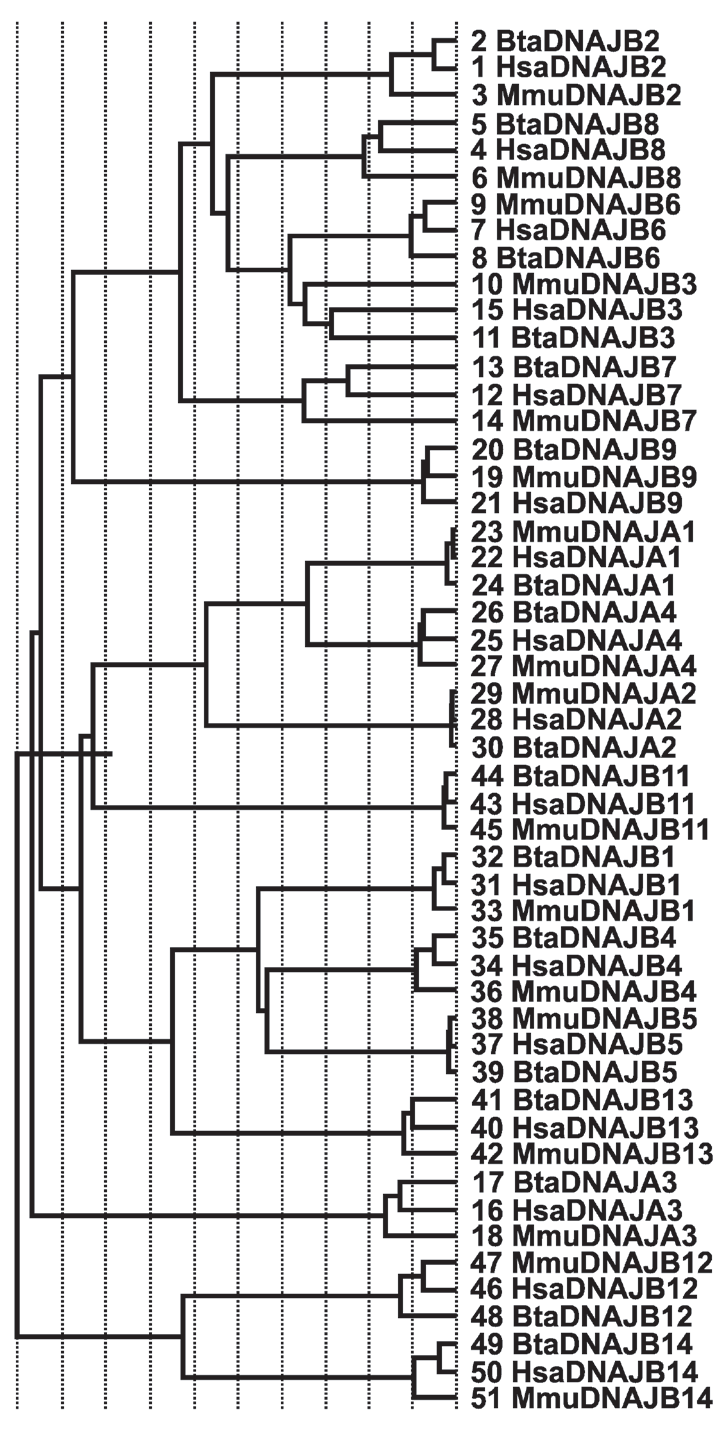
Sequence identity dendrogram of heat shock protein 40 type 1 and 2 gene family members. The first three letters Hsa, Bta and Mmu corresponds to human, bovine and mouse, respectively. The numbers represent the sequence numbers used in the evolutionary trace analysis.

**Figure 7.**
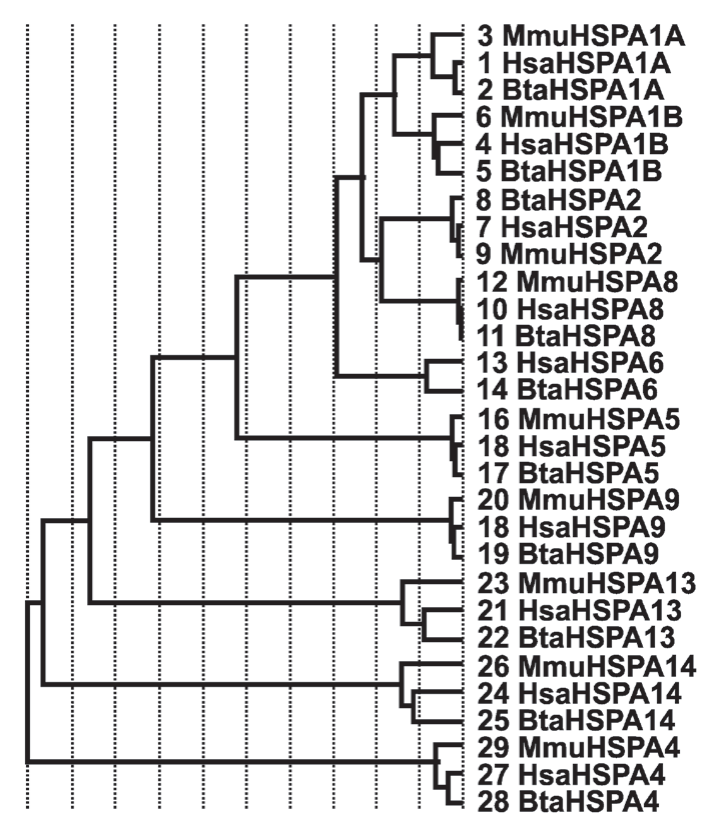
Sequence identity dendrogram of heat shock protein 70 gene family members. The first three letters Hsa, Bta and Mmu corresponds to human, bovine and mouse, respectively. The numbers represent the sequence numbers used in the evolutionary analysis.

**Figure 8.**
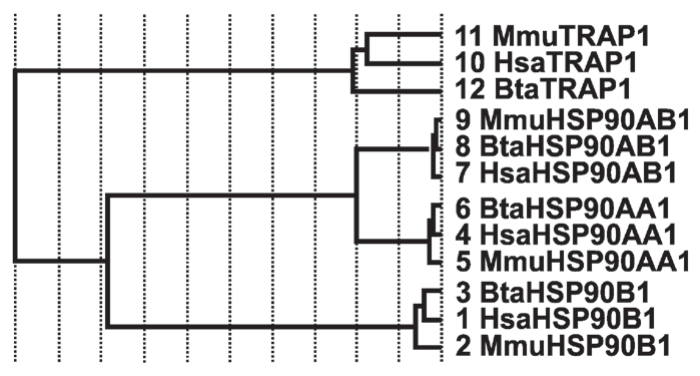
Sequence identity dendrogram of heat shock protein 90 gene family members. The first three letters Hsa, Bta and Mmu corresponds to human, bovine and mouse, respectively. The numbers represent the sequence numbers used in the evolutionary trace analysis.

Sequence identity dendrogram of sHSPs suggests a monophyletic arrangement with *ODF1* diverging first from other members of sHSPs, while *HSPB2*, *CRYAA*, *CRYAB* and *HSPB6* appeared to have recently diverged, with *CRYAA* and *CRYAB* branching from the same node ( Figure 4). In the HSP40 gene family, multiple alignment of consensus sequences were partitioned into seven groups using PDB files 1HDJ and 3AGX as reference structures; this resulted in a trace that identified residues tyrosine (Y), L, A, A, histidine (H), P, F, A, Y, L, R, aspartic acid (D) and G, as evolutionarily conserved residues with some class-specific residues nestled within the J domain ( Figure 5). Sequence identity dendrogram suggest a monophyletic pattern with *DNAJB14* diverging first followed by *DNAJA3* ( Figure 6). While using reference structures (2QW9, 1YUW) for HSP70 genes and (3Q6N, 4AWO) for HSP90 genes, evolutionary trace analysis predicted a large number of amino acid residues to be evolutionarily conserved (data not shown). Sequence identity dendrogram assumed a monophyletic pattern with *HSPA4* and *MT1* diverging first from other members of HSP70 and HSP90 gene families, respectively ( Figure 7, Figure 8).

## Discussion

The highly-conserved heat stress genes are instrumental to maintaining protein homeostasis and coordinating cellular stress responses ( Keller *et al.*, 2008). Our analysis revealed a total of 67 genes (10 sHSP, 43 HSP40, 10 HSP70 and 4 HSP90), which were believed to have occurred because of gene duplication, an event characteristic of many gene families. sHSPs are functionally known to confer protection to a variety of cellular stressors ( Latchman, 2002) and notably involved in cytoskeletal rearrangements ( Quinlan, 2002) and apoptosis ( Arrigo *et al.*, 2002). A search of all bovine genes that code for alpha crystallin-related sHSPs identified 10 sHSP-like proteins in the cattle genome, with the identification of a previously unidentified ODF1, which was newly observed to be present in cattle, but reported in humans ( Kappe *et al.*, 2003) and could possibly be present in other species. In this study, the distribution of sHSP genes in bovines was observed to be dispersed over nine chromosomes ( Table 1) and similar results have been reported in humans ( Kappe *et al*., 2003) suggesting the conservation of sHSP genes in the two species’ common ancestor.

Computational analysis assessing the physico-chemical properties of proteins in the gene families is crucial to understanding the functions of the protein encoded by genes *in vitro*. In this study, the pI was observed to be acidic for most of the cattle sHSPs except for HSPB9 and ODF1, which were basic. These observations might be indicative of functional differences of HSPB9 and ODF1 compared to other members as similar finding might suggest possibly different roles ( Korber *et al.*, 2000). In any case, the *in vivo* functional assessment of these sHSPs in cattle is necessary before making valid conclusions.

In the sHSP gene family, the aliphatic index was high (> 65), implying that cattle sHSP genes possess thermal stability, a feature consistent with its protective function in preventing cellular damage during heat stress ( Collier *et al.*, 2006). Among sHSP genes, GRAVY results suggested that proteins encoded by these genes are hydrophilic, which may enhance its functional ability to oligomerize and its subsequent binding abilities to different proteins ( Lanneau *et al.*, 2008; Roy *et al.*, 2011).

Multiple sequence alignment (MSA) of homologous sequences offers a wealth of information by identifying conserved residues crucial to the function or structure of related proteins ( Capra & Singh, 2007). Evolutionary trace analysis not only helps in identification of evolutionarily conserved residues but also putatively identify functionally important internal and external residues, potentially contributing to cell integrity and enzymatic activity respectively ( Lichtarge *et al.*, 1996). Among cattle sHSP genes, MSA identified most of the evolutionarily conserved residues in the alpha crystallin domain while the amino terminal and carboxylic terminal sequences were deficient of invariant residues. Conservation of the structural architecture of sHSPs in several species has been demonstrated ( Caspers *et al.*, 1995; Kim *et al.*, 1998), with Haslbeck *et al.* (2005) reporting that the N and C termini, though variable in sequence and length, are essential in preventing the misfolding of proteins, this observation neatly validated by our findings.

ET analysis was also utilized in the identification of invariant and class-specific residues and results obtained suggested that a single class-specific residue was observed in the N-terminal region, while four were observed in the alpha crystallin domain when homologous sequences of human, mouse and cattle were included in the ET analysis ( Figure 6). In the alpha crystallin domain, and as revealed by ET results, the exclusion of the distant member *ODF1* gene identified LxxxGxL as one of the conserved motifs shared among human, mouse and cattle sHSP sequences, while residues YxxxSxV are class-specific of the *ODF1* gene. In addition, downstream of the YxxxSxV motif was the presence of approximately 21 residues present only in the *ODF1* gene and this might suggest a structural and functional differences between the *ODF1* gene and other sHSPs in cattle. Interestingly, the sequence identity dendrogram also observed the separation of the *ODF1* gene prior to the divergence of other members of sHSP, thus further strengthening our previous hypothesis of a possible existence of functional and structural differences between ODF1 and other members of the sHSP family. In a related study, several authors reported AxxxxGxL as the most conserved motif in the alpha crystallin domain ( Caspers *et al.*, 1995; Narberhaus, 2002); however, in the MSA of cattle sHSP sequences, it appears that the AxxxxGxL motif has been replaced with LxxxGxL motif. One plausible reason could be that the A residue in cattle may not be essential to cattle sHSPs chaperoning or substrate recognition functions. The two-residue region (LP) observed in extremophiles ( Laksanalamai & Robb, 2004) were also identified in our study upstream the LxxGxL motif, although residue P was identified to be much more highly conserved than residue L ( Figure 6). That said, the *in vivo* roles of these residues remain to be verified, and one useful approach is to carry out an *in-vivo* site-directed mutagenic study.

The HSP40 gene family is a large family that is structurally classified into 3 subtypes ( Cheetham & Caplan, 1998) and functionally characterized based on their role as co-chaperones in binding and regulating the activity of HSP70s ( Jiang *et al.*, 1997). A total of 43 putative HSP40 members were identified in this study and they were scattered across the genome although 41 J-domain containing proteins were reported in humans ( Qiu *et al.*, 2006). The large number of genes identified in the HSP40 family could be adduced to its functional mediatory role in stabilizing the interaction between HSP70 and a myriads of substrates ( Muchowski & Wacker, 2005) in different cellular components to meet cellular goals. GRAVY results of HSP40 suggest hydrophilic tendencies, except for DNAJC22, which appears to be hydrophobic. In addition, while some members appear to be acidic based on their pI values, others possess basic properties thus, suggesting functional differences that could be useful in wet lab experiments.

DNAJ/HSP40 family members contain the J domain, facilitating binding to HSP70s, although other domains have been identified that are critical to their functions ( Kota *et al.*, 2009). MSA results identified evolutionarily conserved residues that are plausibly significant to the overall activity of the J domains or the preservation of its structural integrity. The identification of evolutionarily conserved residues only in the J domain could be consistent with the conservative nature of the J domain in comparison to other domains. The sequence alignment of the HSP40 type I and II homologs in cattle predicted the presence of cysteine repeats which was observed only in DNAJA1, DNAJA2, DNAJA3 and DNAJA4 (HSP40 type I) sequences. This finding as observed in humans, were consistent with the hypothesis that structurally, the presence of the cysteine repeats in HSP40 type I clearly distinguishes it from HSP40 type II and type III ( Shi *et al.*, 2005). The HPD motif present between helix 2 and 3 is reported to mediate the interaction between HSP40 and HSP70 due to the high degree of conservation ( Greene *et al.*, 1998).

In DNAJB13, the cysteine repeats was observed to have been replaced with the HPL motif and this tri-peptide motif was observed to be present in human, mouse and cattle DNAJB13, suggesting that this mutation has occurred before the divergence of these three species. Given the reported functional loss of DNAJ due to H33Q mutation ( Cajo *et al.*, 2006) and SEC63P due to P156N and D157A mutation ( Feldheim *et al.*, 1992), it is unclear whether the motif change from HPD to HPL in DNAJB13 would still enable this gene to perform its HSP70 ATPase activity. That said, the extragenic suppressor analysis of a DNAJ D35N mutant of the HPD motif was reported to cause defective growth and this anomaly was alleviated by the spontaneous mutations of DNAK (hsp70 in *E. coli*) at R167 ( Suh *et al.*, 1998).

ET analysis involving HSP type I and II genes in humans, mice and cattle identified some evolutionarily conserved residues which are consistent with our observations during multiple sequence alignment. Although some class-specific residues were observed, all the invariant residues found in the J domain among orthologous sequences could be suggestive of their functional importance to regulate HSP70 ATPase activity or to ensure protein stability *in vivo*, including tethering DNAK (a member of HSP70) to DNAJ (HSP40)-bound substrates ( Greene *et al.*, 1998). An interesting observation was in the sequence identity dendrogram involving humans, mice and cattle putative HSP40 type I and II sequences. One would have thought that the members of HSP type I ( *DNAJA1*, *DNAJA2*, *DNAJA3*, *DNAJA4*) would have clustered together because of the presence of the four canonical domains; however, *DNAJA3* diverged earlier than expected when compared to other members which recently diverged. In addition, *DNAJA3* did not cluster with any other HSP40 sequences and diverged from the tree after the divergence of the *DNAJB14* and *DNAJB12* clade. Although, the reason for this observation remains unknown at this moment but could be indicative of functional divergence of *DNAJA3*. It therefore appears that *DNAJA3* performs its chaperoning function in a way and manner that is different compared to other HSP40 type 1 and 2 gene family, given the sudden shift of the pI of the DNAJA3 proteome toward basic values when compared to other HSP type I members. In any case, more research needs to be done to functionally verify these speculations.

A total of 10 members of HSP70 were observed in cattle with the invariant residues mostly found in the nucleotide binding domain where HSP70 interacts with HSP40 J domains. Interestingly, isoelectric point (pI) for all the HSP70 protein sequences were all predicted to be acidic with very little variation between the isoelectric point values of HSP70 protein sequences. This could possibly suggest a functional similarity among the cattle HSP70, further confirmed by reports of conserved functional properties of HSP70 protein across species ( Angelidis *et al.*, 1996; Li *et al.*, 1991). All bovine HSP70 protein sequences appear to be hydrophilic based on the GRAVY and the high values predicted in aliphatic index was suggestive of its thermal stability which is consistent with its chaperoning role in achieving HSP70-mediated protection against stresses that causes protein denaturation ( Bukau *et al.*, 2006). In the sequence identity dendogram, it was observed that *HSPA4* diverged first, followed by *HSPA14*, while the instability index was predicted to be unstable (II>40) for HSPA4 (45.24) and HSPA14 (44.95), presumably an indication of functional similarities between HSPA4 and HSPA14; that said, one cannot rule out the fact that one needs to conduct more experiments in order to gain mechanistic insights before valid inferences can be made.

HSP90 is an abundant and highly conserved molecule, whose constitutive forms (HSP90AA1, HSP90AB1, HSP90B1 and the mitochondrial TRAP1) possesses acidic properties. High values were also recorded in the aliphatic index, with the highest value occurring in TRAP1, indicative of high thermal stability when compared to the constitutive forms. Similarly, the stability of HSP90 *in vitro*, as predicted by the instability index and hydrophilic properties inferred from GRAVY results, are useful information that could be utilized in wet lab experiments. The high level of sequence conservation revealed in MSA and ET analysis both in the N- and C-terminal regions suggests these two regions play a crucial role in HSP90 chaperone functions. The sequence identity dendrogram revealed that *TRAP1* diverged first, while the constitutive forms of HSP90 were grouped together in a single clade. This result might be consistent with the fact that TRAP1 primarily functions in the mitochondria while the other members, which makes up the constitutive forms, function in the cytosol.

## Data availability

All GenBank accession numbers of the bovine sequences used in this study are detailed in Table 1– Table 4.
