# Supplementary material for: Computational genome-wide identification of heat shock protein genes in the bovine genome
Source: F1000Res. 2018 Sep 20;7:1504. [Version 1] doi: 10.12688/f1000research.16058.1 (PMC6259560; doi:10.12688/f1000research.16058.1)
Supplement: Peer review history

# Peer review: Referee response for version 1

**DOI:** 10.5256/f1000research.17537.r40818

**Published:** 2018-11-22

**Authors:** Anurag Sharma

**Affiliations:** 1 Indian Institute of Toxicology Research (CSIR-IITR), (CSIR - Council of Scientific & Industrial Research) , Lucknow, Uttar Pradesh, India

In the manuscript entitled ‘Computational genome-wide identification of heat shock protein genes in the bovine genome’, the authors have used *in the silico *approach to identify Hsps in the bovine genome. The authors identified 67 genes belonging to four Hsp families. The manuscript is well written and the observations do support the conclusions.

 However, the authors should address the following concerns:

1. The purpose of the study should be more clear in the introduction.
2. Have the authors checked the Heat Shock Factors similarities with human or mouse?
3. The authors should discuss the functional co-relation of the identified Hsps.

I have read this submission. I believe that I have an appropriate level of expertise to confirm that it is of an acceptable scientific standard.

# Peer review: Referee response for version 1

**DOI:** 10.5256/f1000research.17537.r39863

**Published:** 2018-11-12

**Authors:** Ankit K. Rochani

**Affiliations:** 1 Bio Nano Electronics Research Centre, Graduate School of Interdisciplinary Science, Toyo University, Kawagoe, Japan; 2 Department of Pharmaceutical Science, Jefferson College of Pharmacy, Thomas Jefferson University, Philadelphia, PA, USA

Ajayi *et al.* present an important technically sound *in silico* data about the genes that code for sHsp, Hsp40, Hsp70 and Hsp90 from *Bos Taurus*. It can be accepted as it is after considering the following minor suggestions:

1. Authors are requested to justify the reason for restricting their analysis to only sHsp, Hsp40, Hsp70 and Hsp90.
2. It is unclear why the searches for Hsp90 were unable to identify genes for GRP94.
3. Authors can keep all the identified genes in Tables 1 to 4 in the supplementary figure.
4. Authors are requested to briefly provide comments on the physico-chemical values for the Hsps from bovine in comparison to humans and mouse.

I have read this submission. I believe that I have an appropriate level of expertise to confirm that it is of an acceptable scientific standard.

# Peer review: Referee response for version 1

**DOI:** 10.5256/f1000research.17537.r39552

**Published:** 2018-10-23

**Authors:** José Luis Martínez-Guitarte

**Affiliations:** 1 Grupo de Biología y Toxicología Ambiental, Departamento de Física Matemática y de Fluidos, Universidad Nacional de Educación a Distancia, UNED, Madrid, Spain

In this article Ajayi *et al.* localize 67 different heat shock proteins in the genome of *Bos taurus* using human and mouse proteins and conserved motifs of each family. The proteins identified belong to the small HSP, HSP40, HSP70, and HSP90 groups. The ORF of each protein is analyzed to know its physicochemical properties and an evolutionary study is carried out. The different protein sequences are compared to get conclusions about the conservation and the main characteristics of each set of proteins.

 The approach is adequate but I have some questions. The first one is the source of the *B. taurus* genome. The authors do not explain at any moment which database was used to obtain the genome. Most of the sequences are included in the NCBI protein database, so why did they decide to search directly in the genome before checking the database? On the other hand, they only analyzed the sequences of four families; why did they not include the hsp10, hsp60, and hps110 groups? About the results, it is striking that some proteins that show some of the motifs did not appear in the search. For example, gp96 is a protein with an Hsp90 motif so it is surprising that in a search with Hsp90, Pfam did not give any similarity. Some are similar with other proteins related with the HSP70 family (e.g. HSP12A) due to the presence of an HSP70 motif.

 About the results and discussion, they are mainly focused on the protein sequences and the differences and similarities of amino acids. I miss some discussion related to the gene structure (presence/absence of introns) and related to the transcriptional activity information (what is known from some of the genes described here). I understand that the number of genes is high and it is hard to discuss everything but, in some cases, this information can be helpful in relation to the putative function of the protein. For example, the authors seem to assume that sHSPs are related to stress but many of them have a role in processes like development. It should be taken in account when they are divided into different groups.

 As stated before, I miss some information related to the genes. It would be interesting to incorporate in the tables the position in the chromosomes since the authors have located each gene in the chromosome. In the sHSPs it would be interesting to know if they are close since it has been proposed that duplication is a putative mechanism of increasing the number of members of this family and, often, two related sHSPs are in head-to-head or head-to-tail positions. Also it will be helpful to include the existence of introns in the gene, as it could help to find homologs in other species.

 About the tree, why did the authors not include an example of an external group?

 Overall the article is technically sound and it is a first step to describe more deeply the heat shock protein set in this species. It is important that the authors remark that they are only analyzing four of the families, maybe the most important, but for example, they did not include the Hsp60 and Hsp10 families that are mitochondrial. On the other hand, the main weakness, in my opinion, is to discuss the results without taking into consideration any functional information (especially for sHSPs and HSP70) because it could help to associate putative physicochemical properties with an inducible role.

I have read this submission. I believe that I have an appropriate level of expertise to confirm that it is of an acceptable scientific standard, however I have significant reservations, as outlined above.
